# Experimental evidence for recovery of mercury-contaminated fish populations

**DOI:** 10.1038/s41586-021-04222-7

**Published:** 2021-12-15

**Authors:** Paul J. Blanchfield, John W. M. Rudd, Lee E. Hrenchuk, Marc Amyot, Christopher L. Babiarz, Ken G. Beaty, R. A. Drew Bodaly, Brian A. Branfireun, Cynthia C. Gilmour, Jennifer A. Graydon, Britt D. Hall, Reed C. Harris, Andrew Heyes, Holger Hintelmann, James P. Hurley, Carol A. Kelly, David P. Krabbenhoft, Steve E. Lindberg, Robert P. Mason, Michael J. Paterson, Cheryl L. Podemski, Ken A. Sandilands, George R. Southworth, Vincent L. St Louis, Lori S. Tate, Michael T. Tate

**Affiliations:** 1 Fisheries and Oceans Canada, Freshwater Institute, Winnipeg, Manitoba Canada; 2 Department of Biology, Queen’s University, Kingston, Ontario Canada; 3 IISD Experimental Lakes Area, Winnipeg, Manitoba Canada; 4 Département de Sciences Biologiques, Université de Montréal, Montreal, Quebec Canada; 5 Environmental Chemistry and Technology Program, University of Wisconsin-Madison, Madison, WI USA; 6 Department of Biology, Biological and Geological Sciences Building, University of Western Ontario, London, Ontario Canada; 7 Smithsonian Environmental Research Center, Edgewater, MD USA; 8 Department of Biological Sciences, University of Alberta, Edmonton, Alberta Canada; 9 Department of Biology, University of Regina, Regina, Saskatchewan Canada; 10 Reed Harris Environmental, Oakville, Ontario Canada; 11 University of Maryland Center for Environmental Science, Chesapeake Biological Laboratory, Solomons, MD USA; 12 Water Quality Center, Trent University, Peterborough, Ontario Canada; 13 University of Wisconsin-Madison, Department of Civil and Environmental Engineering, Environmental Chemistry and Technology Program, Madison, WI USA; 14 US Geological Survey, Middleton, WI USA; 15 Oak Ridge National Laboratory, Oak Ridge, TN USA; 16 Department of Marine Sciences, University of Connecticut, Groton, CT USA; 17 Present Address: R&K Research, Salt Spring Island, British Columbia Canada; 18 Present Address: Wisconsin Department of Natural Resources, Madison, WI USA

**Keywords:** Boreal ecology, Ecosystem ecology, Freshwater ecology, Environmental impact

## Abstract

Anthropogenic releases of mercury (Hg)^1–3^ are a human health issue^4^ because the potent toxicant methylmercury (MeHg), formed primarily by microbial methylation of inorganic Hg in aquatic ecosystems, bioaccumulates to high concentrations in fish consumed by humans^5,6^. Predicting the efficacy of Hg pollution controls on fish MeHg concentrations is complex because many factors influence the production and bioaccumulation of MeHg^7–9^. Here we conducted a 15-year whole-ecosystem, single-factor experiment to determine the magnitude and timing of reductions in fish MeHg concentrations following reductions in Hg additions to a boreal lake and its watershed. During the seven-year addition phase, we applied enriched Hg isotopes to increase local Hg wet deposition rates fivefold. The Hg isotopes became increasingly incorporated into the food web as MeHg, predominantly from additions to the lake because most of those in the watershed remained there. Thereafter, isotopic additions were stopped, resulting in an approximately 100% reduction in Hg loading to the lake. The concentration of labelled MeHg quickly decreased by up to 91% in lower trophic level organisms, initiating rapid decreases of 38–76% of MeHg concentration in large-bodied fish populations in eight years. Although Hg loading from watersheds may not decline in step with lowering deposition rates, this experiment clearly demonstrates that any reduction in Hg loadings to lakes, whether from direct deposition or runoff, will have immediate benefits to fish consumers.

## Abstract (web summary)

In a 15-year whole-ecosystem, single-factor experiment, stopping experimental mercury loading results in rapid decreases in methylmercury contamination of fish populations and almost complete recovery within the timeframe of the study.

## Main

The Minamata Convention on Mercury is an international treaty that aims to protect human health and the environment from adverse effects of MeHg by controlling Hg emissions, which should then decrease deposition and loading of anthropogenic Hg to aquatic environments^10^. Yet there is little direct evidence for how quickly fish MeHg concentrations will decline following reductions in current rates of Hg loading owing, in part, to a range of ecological factors that can influence both the microbial production and the bioaccumulation of MeHg in aquatic food webs^9,11^. Further complicating this relationship are human activities such as commercial fishing, introduction of exotic species and enhanced nutrient additions that trigger large-scale trophic disruptions^12^, which can in turn substantially alter fish tissue MeHg concentrations^13–15^, because fish acquire most of their MeHg through their diet^16^. Changing climatic conditions can also influence MeHg production^8^, as well as restructure food webs, alter the dominant pathways of energy flow and cause size-dependent changes in fish growth rates that shift population size structure^14,17,18^. In addition, until now there has been no way to evaluate the relative contribution of newly deposited Hg to contemporary MeHg production. Consequently, it is exceedingly difficult to unambiguously assess the recovery of contaminated fish populations due specifically to Hg control measures^9^.

**Fig. 1:**
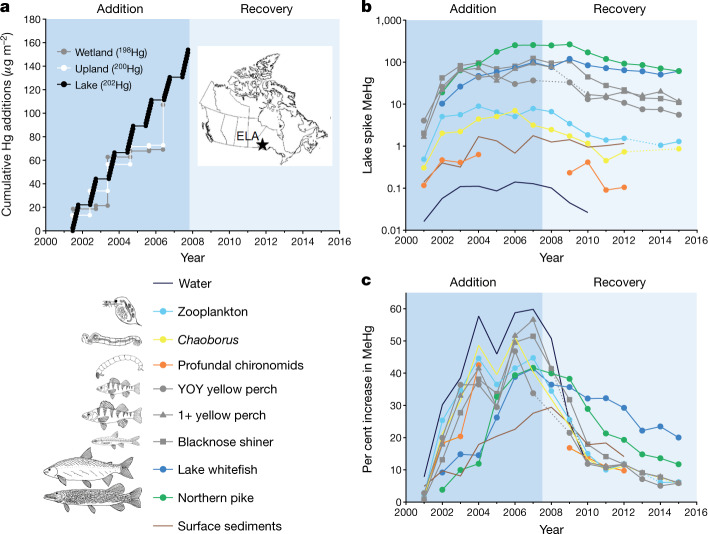
Temporal dynamics of mercury addition and recovery in the Lake 658 ecosystem. **a**, Location (inset) of the Experimental Lakes Area (ELA), Canada, where Hg enriched with different isotopes was applied to the wetland, upland and lake surface of Lake 658 to simulate enhanced wet deposition of Hg (dark blue shaded area). **b**, Inorganic Hg added to the lake was methylated and measured as MeHg concentration in water (in ng l^−1^; *n* = 516), sediments (in ng g^−1^ dry weight; *n* = 1,627) and invertebrates (in ng g^−1^ wet weight; *n* = 211), and as total Hg in fishes (in ng g^−1^ wet weight; *n* = 1,052). Mean annual concentrations for the open-water season are shown for all lake components except for fish populations, which were collected each autumn. Concentration data for large-bodied fish are derived from body-length standardization (pike, 475 mm; whitefish, 535 mm). **c**, Hg loading to the lake increased MeHg concentrations (per cent increase = [lake spike MeHg]/[ambient MeHg] × 100) during the addition phase (2001–2007), then decreased during the recovery phase (2008–2015), when experimental Hg additions to the ecosystem ceased (light blue shaded area in **a**). Dotted lines indicate missing data. Source data.

Over a 15-year period (2001–2015) we conducted a whole-ecosystem Hg loading and recovery experiment (Mercury Experiment To Assess Atmospheric Loading In Canada and the United States (METAALICUS)) in a pristine boreal watershed^19^. METAALICUS addresses the relationship between changes in inorganic Hg loadings to a lake and MeHg concentrations in fish using highly enriched inorganic Hg isotopes (termed ‘spikes’) that enabled us to specifically follow a change in loading against a background of previously deposited Hg and present-day, relatively constant, Hg inputs from direct deposition to the lake surface and from the watershed. In our experiment, these are defined as ‘ambient Hg’. By adding a different spike Hg to the lake (^202^Hg), wetland (^198^Hg) and upland (^200^Hg) compartments of the Lake 658 watershed (52 ha in total) during a 6- to 7-year addition phase (Fig. 1a) we could follow the uptake of MeHg in fish derived solely from newly deposited Hg^19^. We then ceased all experimental additions to determine the magnitude and timing of reductions in fish MeHg concentrations to reductions in Hg loading to the lake, which we tested by tracking the decline in spike MeHg in fish, their prey and other compartments of the lake ecosystem over an eight-year recovery phase. The diverse fish community of the METAALICUS lake enabled assessment of contaminant bioaccumulation and recovery across different trophic guilds and different exposure pathways (that is, sediment versus water) for three species important to freshwater fisheries across the boreal ecoregion^20^ (planktivore: yellow perch (*Perca flavescens*); benthivore: lake whitefish (*Coregonus clupeaformis*); and piscivore: northern pike (*Esox lucius*)).

**Extended Data Fig. 1:**
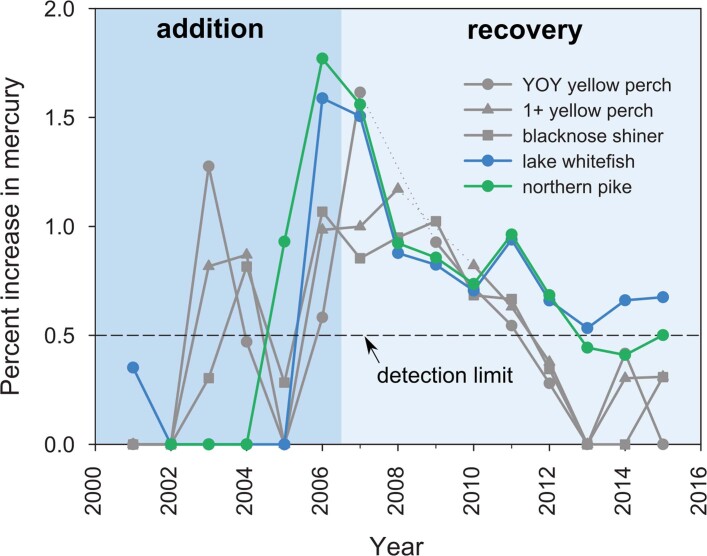
Temporal dynamics of upland mercury in fish. Isotopic ^198^Hg added to the upland area of Lake 658 was above the detection limit (0.5% of ambient MeHg; dashed line) in all fish species, but contributed little (<2%) to overall MeHg concentrations (percent increase = [upland spike MeHg]/[ambient MeHg] × 100). Mean annual MeHg concentration data for each species or age-class is presented and based on fish collected during fall population sampling (*n* = 1,052; sample size details in Extended Data Tables 2 and 3); dotted lines indicate missing data .

The METAALICUS watershed is in an undisturbed remote region of Canada, such that our experimental addition rate increased wet Hg deposition approximately fivefold (from approximately 3.6 to 19 µg m^−2^ yr^−1^), to levels similar to more polluted regions of the world^21^. Most of the Hg added to the wetland and upland areas of the watershed either remained bound to vegetation and soils or evaded back to the atmosphere^22,23^. The wetland spike was below the detection level in all fish species. The Hg applied to the upland catchment accounted for only a small fraction (less than 1%) of all Hg in runoff to the lake^19^ and consequently contributed little (less than 2%) to the changes in MeHg concentrations of fish populations throughout the study (Extended Data Fig. 1). Hence, after six years of increased additions to the watershed (Fig. 1a), the observed increases in fish MeHg were due almost entirely to Hg added directly to the lake surface. This did not appear to be caused by preferential methylation of lake spike Hg. The evidence for this is that the seasonal production of lake spike MeHg, all of which had to be formed within the lake because it was added as inorganic Hg, varied synchronously with ambient MeHg in the lake and in biota^19^. This finding also indicates that in this headwater lake ambient MeHg is mainly derived from in-lake methylation of inorganic ambient Hg, most of which came from the upland catchment.

**Fig. 2:**
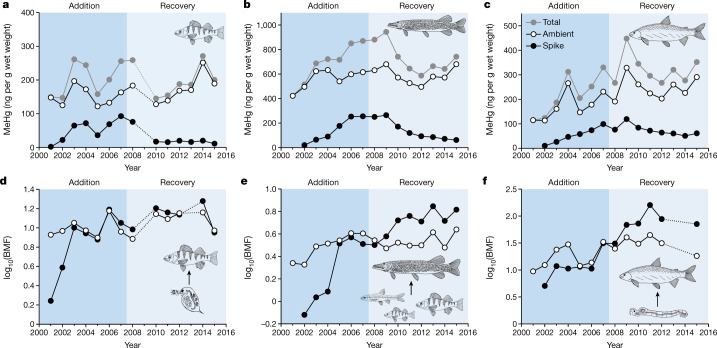
Accumulation and trophic transfer of lake spike and ambient mercury. **a**–**c**, Annual fish muscle MeHg concentrations (total MeHg = lake spike MeHg + ambient MeHg; grey circles) increased above background concentrations (ambient MeHg; white circles) during the addition phase (dark blue shaded area) from uptake of isotope enriched Hg added to Lake 658 (lake spike; black circles) for planktivorous (age 1+ yellow perch; *n* = 140) (**a**), piscivorous (northern pike; *n* = 442) (**b**) and benthivorous (lake whitefish; *n* = 189) (**c**) populations, then declined during the recovery phase (light blue shaded area). **d**–**f**, Biomagnification factors (BMF = [MeHg_predator_]/[MeHg_prey_]) of lake spike MeHg and ambient MeHg from dominant prey items for each of these fish species were as follows: zooplankton (*n* = 127) to yellow perch (**d**); forage fish (*n* = 421) to northern pike (**e**); and *Chaoborus* (*n* = 62) to lake whitefish (**f**). Fish data are means from autumn sampling (sample sizes in Extended Data Tables 2, 3). Concentration data for pike and whitefish are derived from body-length standardization; dotted lines indicate missing data. Source data.

Delivery of lake spike Hg to the sediments and anoxic bottom waters, which are the dominant sites of methylation in the study lake^19,24^, resulted in formation of spike MeHg in these compartments, from where spike MeHg also migrated to surface waters. Lake spike MeHg rapidly accumulated in all lake biota (Fig. 1b), with concentrations in fish muscle increasing with continued loading for all species (Extended Data Table 1; linear regression, *P* < 0.05), apart from young-of-year (YOY) yellow perch, which showed high inter-annual variability in spike MeHg concentrations after an initial increase (Fig. 1b). By contrast, ambient MeHg concentrations in all fish species did not show any consistent trends during the addition phase (Fig. 2a–c), nor did they in a nearby reference lake (Extended Data Table 1; *P* > 0.05). Steady ambient MeHg concentrations in fish through time are indicative of relatively stable watershed inputs of Hg, which is the main source of ambient inorganic Hg for methylation in both the experimental and reference lakes^19,25^.

A critical question for the addition period was how much higher were MeHg concentrations than they would have been in the absence of the experimentally increased loading of Hg. The addition of lake spike Hg was roughly equivalent to all ambient Hg inputs (runoff plus direct deposition) to the lake, resulting in a doubling, or about 100% increase, in Hg loading to the lake^19^. In response to seven years of experimental additions to the lake, per cent increases in lake spike MeHg concentrations were highest in water (60%) and least in the upper 2 cm of sediments (30%) where large stores of ambient Hg existed (Fig. 1c). The response of food web organisms was intermediate to that of water and sediments, such that spike Hg additions to the lake raised MeHg concentrations by 45–57% in invertebrates and forage fishes and by more than 40% for large-bodied fish species (Fig. 1c).

Temporal patterns of biomagnification for spike MeHg relative to ambient MeHg inform how quickly the different fish species came into equilibrium with their respective prey. For small-bodied yellow perch (1 year of age) feeding on zooplankton, it took three years before the biomagnification of lake spike resembled that of ambient MeHg (Fig. 2d), and a further two years for both the apex predator, northern pike, feeding on forage fishes (Fig. 2e), and lake whitefish feeding on *Chaoborus* (Fig. 2f). Relative to planktivorous yellow perch, final addition phase concentrations of spike MeHg were slightly higher for benthivorous lake whitefish (1.2×) and further increased for piscivorous northern pike (3.9×), similar to ambient MeHg (whitefish (1.5×) and pike (3.9×); relative to perch; Fig. 2a–c) and consistent with expectations of contaminant biomagnification among trophic guilds^5,20^. These findings imply that the key in-lake processes leading to the formation and trophic transfer of MeHg to the different fish populations became comparable for spike Hg and ambient Hg during the addition phase.

To then directly test the hypothesis that MeHg concentrations in fishes would decline following reductions in Hg loading to the lake, we ceased all experimental additions of enriched Hg isotopes (Fig. 1a). This resulted in a 100% reduction in loading of lake spike. Average concentrations of lake spike MeHg in fish populations rapidly declined (within less than ten years) in concert with the decline in the availability of spike MeHg through dietary and waterborne pathways (Fig. 1b). Within the first 3 years, the relative amount of lake spike MeHg declined by 81% in water, 35% in sediments, 66% in zooplankton and 67% in *Chaoborus* (Fig. 1c), leading to marked reductions (85–91%) in the concentration of spike MeHg in forage fish species by the end of the recovery phase (Fig. 1b). Eight years after addition, lake spike Hg contributed just a small fraction (approximately 6%) to MeHg concentrations in forage fishes and invertebrate prey (Fig. 1c). The more rapid decline in per cent spike MeHg in water compared with sediments, even in this relatively long water residence time lake (about 6 years), emphasizes that the magnitude and timing of responses by fish to Hg loading reductions could be influenced by their relative reliance upon pelagic versus benthic dietary pathways^26^.

The notably fast response of the lower food web to the cessation of lake spike Hg loadings initiated rapid recovery of large-bodied fish species. Within 8 years, lake spike MeHg concentrations declined by 76% in the northern pike population and by 38% in the lake whitefish population (Fig. 1b). During the recovery phase, spike MeHg concentrations for these large-bodied species initially increased for both populations before showing steady declines (Fig. 2b, c). The rate of decline in spike MeHg for northern pike, however, was roughly twice that of lake whitefish (Fig. 1c).

Differences in the lifespan of the fish populations had a key role in the rates of recovery following the reduction of Hg loadings to the lake. Lake whitefish were much older (median age = 17 years versus 3 years for pike) and larger (Extended Data Tables 2, 3) than northern pike, and more individuals in that population would have lived through some or all of the addition and recovery phases of the experiment. Lake whitefish had the coldest thermal preferences and greatest association with benthic habitats of any fish population, which probably also contributed to their delayed recovery.

**Fig. 3:**
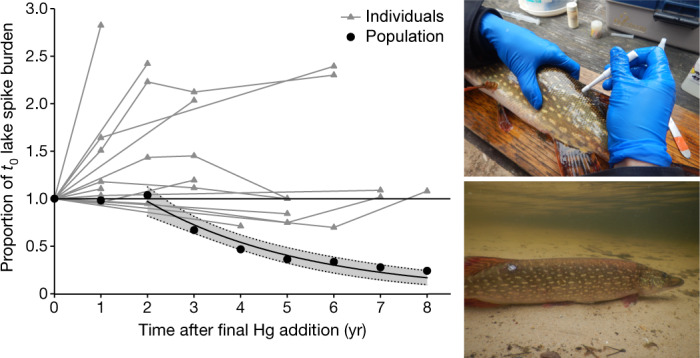
Recovery of the apex predator from mercury loading. Comparison of changes in body burdens of lake spike MeHg during the recovery phase for the northern pike population (annual mean, black circles) to that of individual northern pike (grey lines and triangles). Individual northern pike were sampled at the end of the addition phase (in 2007; *n* = 16) and subsequently recaptured during the recovery phase (each line represents an individual fish). Population data are based on all fish captured each autumn (*n* = 280). All northern pike were sampled using a non-lethal biopsy (represented in images) in the autumn of each year and returned to the lake. Fish body burdens of lake spike MeHg (body burden = lake spike MeHg (ng g^−1^) × fish mass (g)) were normalized to concentrations in the autumn of 2007 (*t*_0_; the final time isotope-enriched Hg was added to Lake 658 and the beginning of the recovery period). Exponential decay regression starting in the second year of recovery estimated a 50% reduction in lake spike MeHg burden in the population in 4.2 years (data are mean (black circle) ± 95% confidence interval (shaded band); line fit: *y* = 1.7439 × e^−0.2928*x*^, *R*^2^ = 0.95, *F*_1,6_ = 95.5, *P* = 0.0002). Source data.

**Extended Data Fig. 2:**
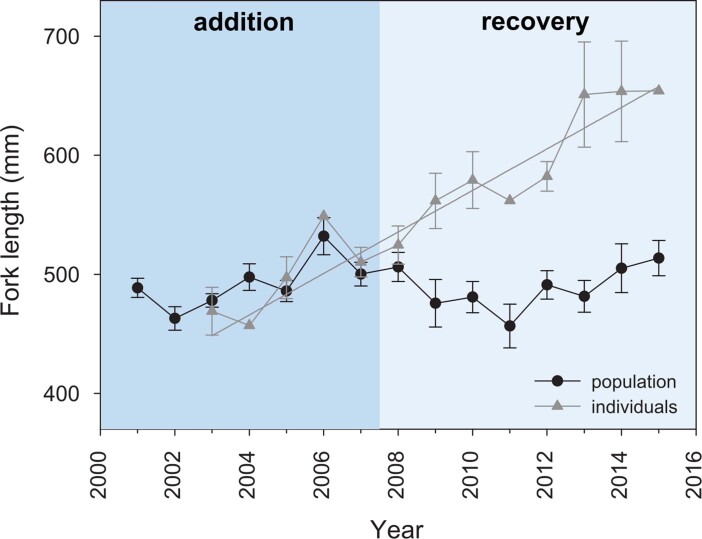
Comparison of individual and population body sizes of northern pike. Mean (± s.e.m.) body size of all northern pike sampled in the fall of each year (population, black circles; *n* = 442) for muscle MeHg concentration using a biopsy method was stable over time. Individual northern pike (grey triangles) captured in 2007 and again in at least one subsequent year (*n* = 16 fish with 1–9 individuals recaptured each year 2008–2015) were used to determine individual losses of lake spike MeHg during recovery (see Fig. 3). These individual fish, which were also captured prior to 2007, showed an increase in body size over time (linear regression: *y* = 17.43*x*−34470.0, *R*^*2*^ = 0.55, *F*_*1,51*_ = 61.1, *P* < 0.0001) .

Boreal fishes are known to eliminate MeHg very slowly once accumulated^27^. To further explain the recovery of the apex predator population, we tracked changes in the body burdens of spike MeHg in individual northern pike over time while also monitoring the population as a whole. As expected, individual responses were variable, but lake spike MeHg burdens in northern pike mostly increased during the early recovery phase with overall little to no loss of the spike MeHg 6–8 years after cessation of spike additions (Fig. 3). These findings parallel those observed for lake spike MeHg in individual northern pike moved from the study lake to a nearby reference lake^28^ and underscore how the prolonged retention of MeHg in fish muscle tissue can delay recovery of some fisheries^7,29^. Thus, it was the annual recruitment of new fish with low MeHg concentrations into the population, along with the loss of older fish (as evidenced by a stable population size structure; Extended Data Fig. 2), that enabled the swift recovery of the population from Hg contamination as a whole. Consequently, average burdens of spike MeHg in the northern pike population were reduced by 50% in less than five years, in spite of the efficient retention of spike MeHg by some older fish (Fig. 3, Extended Data Fig. 2).

Differentiating the relative importance of present day inputs versus previously deposited Hg to overall fish MeHg concentrations is a key uncertainty when predicting the efficacy of Hg pollution reduction^29,30^. Here we demonstrate that within a few years of abatement, the experimental Hg added previously to the lake was no longer an important source of MeHg to the lower food web or to forage fishes (Fig. 1c). Long-lived fish species of subsistence, commercial and recreational importance lagged behind their prey, but the contribution of recently deposited Hg to fish MeHg steadily diminished for these populations as well. There was a similar, rapid response for the upland spike when loading ceased, even though only a small amount appeared in the fish during the experiment (Extended Data Fig. 1). The small contribution of the terrestrial spike to fish MeHg supports our former conclusion^19^ that lakes with large watersheds will respond more slowly to changes in atmospheric deposition.

The most important outcome of this whole-ecosystem experiment is the demonstration that a decrease in a single factor (Hg loading to the lake) has a clear and timely effect on average MeHg concentrations in fish populations, even for long-lived species that eliminate MeHg slowly. The spike MeHg data show that fish populations will respond quickly to any change in loading rates—whether from direct deposition to the lake (Fig. 1) or runoff (Extended Data Fig. 1). Decreases in loading to the lake from these two sources will follow different time courses in response to lower atmospheric deposition^19^. However, as these two loads decrease, the fish populations in the receiving lake will soon afterwards have lower MeHg than they would have if nothing were done, thereby reducing human exposure.

## Methods

### Mercury additions to the study catchment

METAALICUS was conducted on the Lake 658 catchment at the Experimental Lakes Area (ELA; now IISD-ELA), a remote area in the Precambrian Shield of northwestern Ontario, Canada (49° 43′ 95″ N, 93° 44′ 20″ W) set aside for whole-ecosystem research^31^. The Lake 658 catchment includes upland (41.2 ha), wetland (1.7 ha) and lake surface (8.4 ha) areas. Lake 658 is a double basin (13 m depth), circumneutral, headwater lake, with a fish community consisting of forage (yellow perch (*P. flavescens*) and blacknose shiner (*Notropis heterolepis*)), benthivorous (lake whitefish (*C. clupeaformis*) and white sucker (*Catostomus commersonii*)), and piscivorous (northern pike (*E. lucius*)) fishes. The lake is closed to fishing.

Hg addition methods used in METAALICUS have been described in detail elsewhere^19,32,33^. In brief, three Hg spikes, each enriched with a different stable Hg isotope, were applied separately to the lake surface, upland and wetland areas. Upland and wetland spikes were applied once per year (when possible; Fig. 1a) by fixed-wing aircraft (Cessna 188 AGtruck). Mercury spikes (as HgNO_3_) were diluted in acidified water (pH 4) in a 500 l fiberglass tank and sprayed with a stainless-steel boom on upland (approximately 79.9% ^200^Hg) and wetland (approximately 90.1% ^198^Hg) areas. Spraying was completed during or immediately before a rain event, with wind speeds less than 15 km h^−1^ to minimize drift of spike Hg outside of target areas. Aerial spraying of upland and wetland areas left a 20-m buffer to the shoreline, which was sprayed by hand with a gas-powered pump and fire hose to within about 5 m of the lake^32^. Average net application rates of isotopically labelled Hg to the upland and wetland areas were 18.5 μg m^−2^ yr^−1^ and 17.8 μg m^−2^ yr^−1^, respectively.

The average net application rate for lake spike Hg was 22.0 μg m^−2^ yr^−1^. For each lake addition, inorganic Hg enriched with approximately 89.7% ^202^Hg was added as HgNO_3_ from four 20-l carboys filled with acidified lake water (pH 4). Nine lake additions were conducted bi-weekly at dusk over an 18-week (wk) period during the open-water season of each year (2001–2007) by injecting at 70-cm depth into the propeller wash of trolling electric motors of two boats crisscrossing each basin of the lake^32,33^. It was previously demonstrated with ^14^C additions to an ELA lake that this approach evenly distributed spike added in the evening by the next morning^34^.

We did not attempt to simulate Hg in rainfall for isotopic lake additions because it is impossible to simulate natural rainfall concentrations (about 10 ng l^−1^) in the 20-l carboys used for additions. Instead, our starting point for the experiment was to ensure that the spike was behaving as closely as possible to ambient surface water Hg very soon after it entered the lake. Several factors support this assertion. By the next morning each spike addition had increased epilimnetic Hg concentrations by only 1 ng l^−1 202^Hg. Average ambient concentrations were 2 ng l^−1^. Thus, while the Hg concentrations in the carboys were high (2.6 mg l^−1^), the receiving waters were soon at trace levels. Furthermore, we investigated if the additions altered the degree of bioavailability or photoreactivity of Hg(ii) in the receiving surface water. We examined the bioavailability of spike Hg(ii) as compared to ambient Hg in the lake itself using a genetically engineered bioreporter bacterium^35^. On seven occasions, epilimnetic samples were collected on the day before and within 12 h of spike additions. The spike was added to the lake as Hg(NO_3_)_2_, which is bioavailable to the bioreporter bacterium (detection limit = 0.1 ng Hg(ii) l^−1^), but we never saw bioavailable ambient or spike Hg(ii) in the lake, presumably because it was quickly bound to dissolved organic carbon (DOC). This indicates that, in terms of bioavailability, the spike Hg was behaving like ambient Hg soon after additions. Photoreactivity in the surface water was examined on seven occasions, by measuring the % of total Hg(ii) that was dissolved gaseous Hg for spike and ambient Hg, either 24 h or 48 h after the lake was spiked^36^. There was no significant difference (paired *t*-test, *P* > 0.05), demonstrating that by then the lake spike was behaving in the same way as ambient Hg during gaseous Hg production.

### Lake, food web and fish sampling

Water samples were collected from May to October every four weeks at the deepest point of Lake 658. Water was pumped from six depths through acid-cleaned Teflon tubing into acid-cleaned Teflon or glass bottles. Water samples were filtered in-line using pre-ashed quartz fibre filters (Whatman GFQ, 0.7 µm). Subsequently, Hg species were measured in the filtered water samples (dissolved Hg and MeHg) and in particles collected on the quartz fibre filter (particulate Hg and MeHg).

From 2001 to 2012, Lake 658 sediments were sampled at 4 fixed sites up to 5 times per year. Sampling frequency was highest in 2001, with monthly sampling from May to September, and declined over the course of the study. Fixed sites were located at depths of 0.5, 2, 3 and 7 m. A sediment survey of up to 12 additional sites was also conducted once or twice each year. Survey sites were selected to represent the full range of water depths in both basins. Cores were collected by hand by divers, or by subsampling sediments collected using a small box corer. Cores were capped and returned to the field station for processing within a few hours. For each site, three separate cores were sectioned and composited in zipper lock bags for a 0- to 2-cm depth sampling horizon, and then frozen at −20 °C.

Bulk zooplankton and *Chaoborus* samples were collected from Lake 658 for MeHg analysis. Zooplankton were collected during the day from May to October (bi-weekly: 2001–2007; monthly: 2008–2015). A plankton net (150 μm, 0.5 m diameter) was towed vertically through the water column from 1 m above the lake bottom at the deepest point to the surface of the lake. Samples were frozen in plastic Whirl-Pak bags after removal of any *Chaoborus* using acid-washed tweezers. Dominant zooplankton taxa in Lake 658 included calanoid copepods (*Diaptomus oregonensis*) and Cladocera (*Holopedium glacialis*, *Daphnia pulicaria* and *Daphnia mendotae*). *Chaoborus* samples were collected monthly in the same manner at least 1 h after sunset. After collection, *Chaoborus* were picked from the sample using forceps and frozen in Whirl-Pak bags. *Chaoborus* were not separated by species for MeHg analyses, but both *C. flavicans* and *C. punctipennis* occur in the lake. Profundal chironomids were sampled at the deepest part of the lake using a standard Ekman grab sampler. Grab material was washed using water from a nearby lake and individual chironomids were picked by hand.

All work with vertebrate animals was approved by Animal Care Committees (ACC) through the Canadian Council on Animal Care (Freshwater Institute ACC for Fisheries and Oceans Canada, 2001–2013; University of Manitoba ACC for IISD-ELA, 2014–2015). Licenses to Collect Fish for Scientific Purposes were granted annually by the Ontario Ministry of Natural Resources and Forestry. Prior to any Hg additions, a small-mesh fence was installed at the outlet of Lake 658 to the downstream lake to prevent movement of fish between lakes. Sampling for determination of MeHg concentrations (measured as total mercury (THg), see below) occurred each autumn (August–October; that is, the end of the growing season in north temperate lakes) for all fish species in Lake 658, and for northern pike and yellow perch in nearby reference Lake 240 (Extended Data Tables 2, 3). Fish collections occurred randomly throughout the lakes. Forage fish (YOY and 1+ yellow perch, and blacknose shiner) were captured using small mesh gillnets (6–10 mm) set for <20 min, seine nets, and hoop nets. A small number of fish (up to *n* = 20) of each species and age class (determined by visual inspection) were euthanized immediately following capture in an overdose bath of 0.25 g l^−1^ tricaine methanesulfonate (TMS; Syndel Laboratories). After transport to the field station, fish were measured for fork length (FL; in mm) and mass (to 0.1 g), then immediately frozen (at −20 °C) in individual WhirlPak bags. A year class failure of yellow perch resulted in a single YOY collected in 2008 (data not presented) and no age 1+ fish in 2009 (Extended Data Table 3).

Large-bodied fish were captured by angling and multi-mesh gill nets (2.5–11.4 cm mesh) set for 20–30 min. Upon capture, each fish was anaesthetized with 0.06 g l^−1^ TMS, measured for FL (mm), weighed (to 1 g), tagged (Passive Integrated Transponder; Biomark), and a small biopsy of dorsal muscle (0.091 ± 0.002 g wet weight (mean ± s.e.m)) was collected using a dermal punch^37^. Only fish large enough for the biopsy procedure were sampled, such that our analyses include very few juveniles (pike: 317–850 mm FL; whitefish: 344–874 mm FL). Muscle samples were inserted into 0.6-ml polypropylene vials (Rose Scientific), immediately put on ice, and frozen within 4 h (−20 °C). This non-lethal method permitted repeated sampling of individual fish over time^28,37^. The first ray of either the pectoral or pelvic fin was collected for aging purposes upon first capture. Fish recovered from anaesthesia in a tub of fresh lake water (~15 min) before being released back into the lakes. From 2001–2015, we collected 690 biopsy muscle samples from 390 fish (238 northern pike, 114 lake whitefish and 38 white sucker) in Lake 658; 149 fish (90 northern pike, 38 lake whitefish and 21 white sucker) were biopsied more than once (2 to 6 per individual). Because of consistently low annual catches of white sucker (<10 individuals) across sampling years, we have excluded them from our analyses, but note here that their patterns of lake spike MeHg accumulation and recovery were similar to those of lake whitefish. We were unable to sex most fish because they were either immature or captured outside of their spawning season.

### Sample processing and analytical methods

Detailed methods on sample preparation and MeHg or THg analysis, as well as interlaboratory calibrations, have been reported elsewhere for the METAALICUS project^19,38,39^. In brief, MeHg was distilled from water samples and from sediment using atmospheric pressure water vapour distillation and measured after aqueous phase ethylation using sodium tetraethylborate (NaBEt_4_). Volatile Hg species were purged and trapped onto Tenax and MeHg was measured after thermodesorption and GC separation using inductively coupled plasma mass spectrometry (ICP-MS) detection (Micromass Platform or Perkin-Elmer Elan DRC II, respectively)^39^ and quantification by species specific isotope dilution mass spectrometry. The MeHg isotope dilution standards were synthesized and calibrated in-house. Isotope-dilution spikes were added prior to distillation, and MeHg external standards were routinely calibrated against degradation by measuring the standard against inorganic Hg before and after BrCl digestion. The QC strategy include the regular analysis of blanks, laboratory duplicates and certified reference materials (CRMs) IAEA 405 (International Atomic Energy Agency, Vienna, Austria) and NIST 1566b (National Institute of Standards and Technology, Gaithersburg, Maryland) for MeHg. No CRMs are commercially available for MeHg in water.

All biota samples were handled using clean techniques with Teflon or stainless steel tools cleaned with 95% ethanol^19,38^. Zooplankton and *Chaoborus* were freeze dried, ground with an acid-washed mortar and pestle, subsampled, and weighed to the nearest 0.00001 g. For determination of MeHg concentrations (ambient, lake spike, upland spike and wetland spike) in invertebrate samples, MeHg was solubilized by treatment with a solution of KOH in ethanol (20 % w/v), ethylated by additions of NaBEt_4_, and the resulting volatile Hg species were purged and trapped on carbotrap^39^. Samples were thermally desorbed and separated by gas chromatography before quantification by ICP-MS as above^39^. Samples of CRMs (TORT2 (2001–2013), IAEA452 (2014–2015); National Research Council of Canada, Ottawa, Ontario) were subjected to the same procedures; measured MeHg concentrations in the reference materials were not statistically different from certified values (*P* > 0.05).

Prey fish were kept frozen to maintain consistent wet weights. Approximately 0.2 g of skinless dorsal muscle was removed from each fish, weighed (0.0001 g), and placed in an acid-washed glass vial with a Teflon-lined cap (National Scientific Company). Muscle biopsy samples were weighed to the nearest 0.00001 g (Sartorius BP211D, Data Weighing Systems) before and after freeze-drying (Lyph-lock 12-l freeze dry system Model 77545, Labconco) to obtain wet and dry sample masses, and dry weight proportion^28^. Fish samples were analysed for THg, which is the sum of organic and inorganic Hg. Because we had previously determined that >90% of the Hg in muscle tissue from yellow perch in Lake 658 is MeHg^40,41^, here we report fish mercury data as MeHg.

THg concentrations (ambient, lake spike, upland spike and wetland spike) in fish muscle samples were quantified by ICP-MS^39^. Samples were digested with HNO_3_/H_2_SO_4_ (7:3 v/v) and heated at 80 °C until brown NOx gases no longer formed. The THg in sample digests was reduced by SnCl_2_ to Hg^0^ which was then quantified by ICP-MS (Thermo-Finnigan Element2) using a continuous flow cold vapour generation technique^41^. To correct for procedural recoveries, all samples were spiked with ^201^HgCl_2_ prior to sample analysis. Samples of CRMs (DORM2 (2001–2011), DORM3 (2012–2013), DORM4 (2014–2015); National Research Council of Canada) were submitted to the same procedures; measured THg concentrations in the reference materials were not statistically different from certified values (*P* > 0.05). Detection limit for each of the spikes was 0.5% of ambient Hg.

### Calculations and statistical methods

Analyses were completed with Statistica (6.1, Statsoft) and Sigmaplot (11.0, Systat Software). We present wet weight (w.w.) MeHg concentrations for all samples, except sediments which are dry weight (d.w.) concentrations. For zooplankton, *Chaoborus*, and profundal chironomids, d.w. MeHg concentrations were multiplied by a standard proportion (0.15) to yield w.w. concentrations for each sample^42^. The resulting w.w. concentrations were averaged over each open water season to determine annual means. For fish muscle biopsies, d.w. MeHg concentrations were multiplied by individual d.w. proportions to yield w.w. MeHg concentrations for each sample. To avoid any size-related biases, we calculated standardized annual MeHg concentrations (ambient and lake spike) for northern pike and lake whitefish by determining best-fit relationships between FL and MeHg concentrations for each year (quadratic polynomial, except for a linear fit for lake whitefish in 2004), and using the resulting regression equations to estimate MeHg concentrations at a standard FL^43^ (the mean FL of all fish sampled for each species: northern pike, 475 mm; lake whitefish, 530 mm). Square root transformation of raw northern pike data was required to satisfy assumptions of normality and homoscedasticity prior to standardization. The resulting data represent standardized concentrations of lake spike and ambient MeHg for each species each year.

We used the ratio of lake spike and ambient Hg in each sample as a measure of the amount by which Hg concentrations were changed with the addition of isotopically enriched Hg:

1 $${\rm{P}}{\rm{e}}{\rm{r}}{\rm{c}}{\rm{e}}{\rm{n}}{\rm{t}}\,{\rm{i}}{\rm{n}}{\rm{c}}{\rm{r}}{\rm{e}}{\rm{a}}{\rm{s}}{\rm{e}}={[{\rm{l}}{\rm{a}}{\rm{k}}{\rm{e}}{\rm{s}}{\rm{p}}{\rm{i}}{\rm{k}}{\rm{e}}{\rm{H}}{\rm{g}}]}_{i}/{[{\rm{a}}{\rm{m}}{\rm{b}}{\rm{i}}{\rm{e}}{\rm{n}}{\rm{t}}{\rm{H}}{\rm{g}}]}_{i}\times 100$$

where [lake spike Hg]_*i*_ is the concentration of lake spike MeHg in sample *i*, and [ambient Hg]_*i*_ is the concentration of ambient MeHg in sample *i*. For northern pike and lake whitefish, we calculated the mean annual relative increase from all individuals (not the size-standardized concentration data).

Biomagnification factors (BMF) were calculated to describe differences in Hg concentrations between predator and prey^5^:

2 $${\rm{BMF}}={\log }_{10}({[{\rm{MeHg}}]}_{{\rm{p}}{\rm{r}}{\rm{e}}{\rm{d}}{\rm{a}}{\rm{t}}{\rm{o}}{\rm{r}}}/{[{\rm{MeHg}}]}_{{\rm{p}}{\rm{r}}{\rm{e}}{\rm{y}}})$$

where [MeHg]_predator_ is the mean (forage fish) or standardized (large-bodied fish) concentration of MeHg in the predator (ng g^−1^ w.w.) and [MeHg]_prey_ is the mean concentration of MeHg in the prey (ng g^−1^ w.w.). MeHg concentration of prey items were averaged from samples collected throughout the open-water season immediately prior to autumn sampling of fish species to represent an integrated exposure for calculation of BMF. We used a dominant prey item to represent the diet of each fish species. For age 1+ yellow perch, northern pike, and lake whitefish, dominant prey items were zooplankton, forage fishes (YOY and 1+ yellow perch, and blacknose shiner) and *Chaoborus*, respectively.

To assess loss of lake spike MeHg by northern pike during the recovery period (2008–2015), we calculated^28^ whole body burdens (in μg) of lake spike MeHg for the standardized population and for individuals that had been sampled in autumn 2007 (*t*_0_ is the final time spike Hg was added to the lake) and again in at least one subsequent year during annual autumn sampling (*n* = 16 fish, of which 1–9 individuals were recaptured annually from 2008–2015). This calculation of MeHg burden is a relative measure of whole fish Hg content because MeHg is higher in muscle tissue than in other tissue types^28,40^. For the standardized population data, we used best-fit relationships between FL (in mm) and body weight (in g; quadratic polynomial) to determine body weight at the standard FL. We multiplied this body weight by standard ambient and spike MeHg concentrations (in ng g^−1^ w.w.) in muscle tissue for each year to determine body burdens over time (in ng). For individual fish, we multiplied spike MeHg concentration (in ng g^−1^ w.w.) by body weight (in g) to yield individual body burdens (in ng). To account for differences among individuals and between individuals and the population, we normalized the data to examine the mean proportion of original (*t*_0_) lake spike MeHg burden present in northern pike each year of the recovery period (2008–2015).

3 $${\rm{change}}\,{\rm{in}}\,{\rm{burden}}\,{\rm{from}}\,{t}_{0}={{\rm{burden}}}_{{\rm{tx}}}/{{\rm{burden}}}_{{\rm{t}}0}$$

We used a best fit regression (exponential decay, beginning in the second year of recovery) to estimate the half-life (50% of original burden) of lake spike MeHg for the population.

Northern pike and lake whitefish ages were determined by cleithra and otoliths, respectively, if mortality had occurred, but most ages were quantified using fin rays collected from live fish^44^ (K. H. Mills, DFO or North/South Consultants). Northern pike of the sizes selected for biopsy sampling had a median age of 3 years (range: 2–12 years; *n* = 305); the median age of lake whitefish was 17 years (range: 3–38 years; *n* = 86).

### Reporting summary

Further information on research design is available in the Nature Research Reporting Summary linked to this paper.

## Online content

Any methods, additional references, Nature Research reporting summaries, source data, extended data, supplementary information, acknowledgements, peer review information; details of author contributions and competing interests; and statements of data and code availability are available at 10.1038/s41586-021-04222-7.

### Supplementary information

Reporting Summary

Peer Review File

### Source data

Source Data Fig. 1

Source Data Fig. 2

Source Data Fig. 3

Source Data Extended Data Fig. 1

Source Data Extended Data Fig. 2

## Figures and Tables

**Extended Data Table 1:**
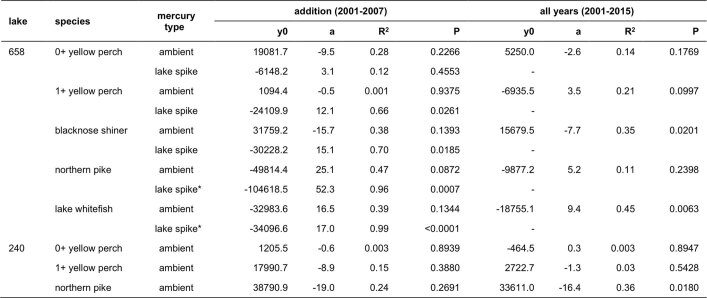
Fish mercury concentrations in the experimental and reference lakes over time

Footnote: Linear regression statistics [*F* = *y*0 + (a × *x*)] of the change in annual mean (yellow perch, blacknose shiner) or body-size standardized (northern pike, lake whitefish) concentrations (ng g^−1^ w.w.) of ambient and lake spike MeHg (measured as THg) in fish muscle tissue during the addition phase or entire study. Isotopic Hg additions were made to Lake 658 from 2001–2007; Lake 240 is a reference lake. Fish collections occurred each autumn.

*lake spike below detection in 2001 (pike: *n* = 32; whitefish: *n* = 7 of 9); regression from 2002–2007

**Extended Data Table 2:**
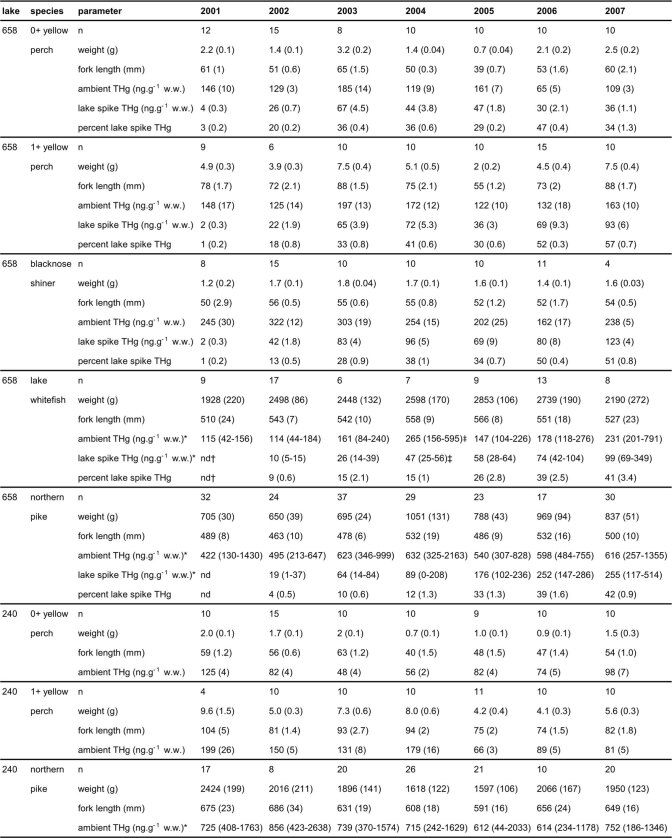
Annual fish metrics and mercury concentrations during the addition phase

Footnote: Data presented are mean (± s.e.m.) of *n* fish measured and their muscle tissue analysed for ambient and lake spike MeHg concentrations (ng g^−1^ w.w., measured as THg), except for northern pike and lake whitefish where concentration data were standardized by fish length (range in parentheses). Percent lake spike THg = [lake spike THg]_*i*_/[ambient THg]_*i*_ × 100. Isotopic Hg additions were made to Lake 658 from 2001–2007; Lake 240 is a reference lake. Fish collections occurred each autumn.

nd = not detected (detection limit for lake spike is 0.5% of ambient THg).

*Standardized by fish length (pike: 475 mm; whitefish: 535 mm).

†Two fish above detection limit.

‡Standardized with linear regression instead of polynomial.

**Extended Data Table 3:**
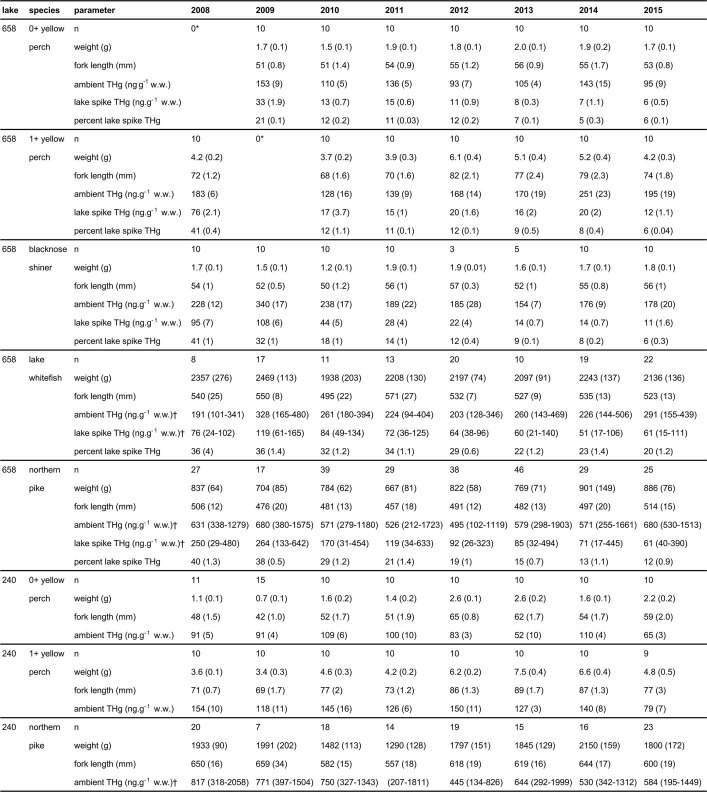
Annual fish metrics and mercury concentrations during the recovery phase

Footnote: Data presented are mean (± s.e.m.) of *n* fish measured and their muscle tissue analysed for ambient and lake spike MeHg concentrations (ng g^−1^ w.w., measured as THg), except for northern pike and lake whitefish where concentration data were standardized by fish length (range in parentheses). Percent lake spike THg = [lake spike THg]_*i*_/[ambient THg]_*i*_ × 100. Isotopic Hg additions were made to Lake 658 from 2001–2007; Lake 240 is a reference lake. Fish collections occurred each autumn.

*Year class failure.

†Standardized by fish length (pike: 475 mm; whitefish: 535 mm).

## Data Availability

Datasets generated in this study are available at 10.5061/dryad.nzs7h44sf. Source data are provided with this paper.
