# Supplementary material for: Experimental evidence for recovery of mercury-contaminated fish populations
Source: Nature. 2021 Dec 15;601(7891):74–8. doi: 10.1038/s41586-021-04222-7 (PMC8732272; doi:10.1038/s41586-021-04222-7)
Supplement: Supplementary file 1 — Reporting Summary [file 41586_2021_4222_MOESM1_ESM.pdf]

nature portfolio

Corresponding author(s): *Paul Blanchfield*

Last updated by author(s): *Sep 29, 2021*

Reporting Summary

Nature Portfolio wishes to improve the reproducibility of the work that we publish. This form provides structure for consistency and transparency in reporting. For further information on Nature Portfolio policies, see our Editorial Policies and the Editorial Policy Checklist.

Statistics

For all statistical analyses, confirm that the following items are present in the figure legend, table legend, main text, or Methods section.

n/a	Confirmed	
☐	☒	The exact sample size (n) for each experimental group/condition, given as a discrete number and unit of measurement
☐	☒	A statement on whether measurements were taken from distinct samples or whether the same sample was measured repeatedly
☐	☒	The statistical test(s) used AND whether they are one- or two-sided<br>*Only common tests should be described solely by name; describe more complex techniques in the Methods section.*
☒	☐	A description of all covariates tested
☐	☒	A description of any assumptions or corrections, such as tests of normality and adjustment for multiple comparisons
☐	☒	A full description of the statistical parameters including central tendency (e.g. means) or other basic estimates (e.g. regression coefficient) AND variation (e.g. standard deviation) or associated estimates of uncertainty (e.g. confidence intervals)
☐	☒	For null hypothesis testing, the test statistic (e.g. F, t, r) with confidence intervals, effect sizes, degrees of freedom and P value noted<br>*Give P values as exact values whenever suitable.*
☒	☐	For Bayesian analysis, information on the choice of priors and Markov chain Monte Carlo settings
☒	☐	For hierarchical and complex designs, identification of the appropriate level for tests and full reporting of outcomes
☒	☐	Estimates of effect sizes (e.g. Cohen's d, Pearson's r), indicating how they were calculated

Our web collection on statistics for biologists contains articles on many of the points above.

Software and code

Policy information about availability of computer code

Data collection	*No software was used.*
Data analysis	*Statistica v 6.1 (Statsoft Inc.); SigmaPlot v11.0 (Systat Software Inc.); Microsoft Excel (2010)*

For manuscripts utilizing custom algorithms or software that are central to the research but not yet described in published literature, software must be made available to editors and reviewers. We strongly encourage code deposition in a community repository (e.g. GitHub). See the Nature Portfolio guidelines for submitting code & software for further information.

Data

Policy information about availability of data

All manuscripts must include a data availability statement. This statement should provide the following information, where applicable:
- Accession codes, unique identifiers, or web links for publicly available datasets
- A description of any restrictions on data availability
- For clinical datasets or third party data, please ensure that the statement adheres to our policy

Datasets generated in this study are available at (doi:10.5061/dryad.nzs7h44sf) and are provided as Source data.

Field-specific reporting

Please select the one below that is the best fit for your research. If you are not sure, read the appropriate sections before making your selection.

☐ Life sciences ☐ Behavioural & social sciences ☒ Ecological, evolutionary & environmental sciences

For a reference copy of the document with all sections, see nature.com/documents/nr-reporting-summary-flat.pdf

Life sciences study design

All studies must disclose on these points even when the disclosure is negative.

Sample size	*Describe how sample size was determined, detailing any statistical methods used to predetermine sample size OR if no sample-size calculation was performed, describe how sample sizes were chosen and provide a rationale for why these sample sizes are sufficient.*
Data exclusions	*Describe any data exclusions. If no data were excluded from the analyses, state so OR if data were excluded, describe the exclusions and the rationale behind them, indicating whether exclusion criteria were pre-established.*
Replication	*Describe the measures taken to verify the reproducibility of the experimental findings. If all attempts at replication were successful, confirm this OR if there are any findings that were not replicated or cannot be reproduced, note this and describe why.*
Randomization	*Describe how samples/organisms/participants were allocated into experimental groups. If allocation was not random, describe how covariates were controlled OR if this is not relevant to your study, explain why.*
Blinding	*Describe whether the investigators were blinded to group allocation during data collection and/or analysis. If blinding was not possible, describe why OR explain why blinding was not relevant to your study.*

Behavioural & social sciences study design

All studies must disclose on these points even when the disclosure is negative.

Study description	*Briefly describe the study type including whether data are quantitative, qualitative, or mixed-methods (e.g. qualitative cross-sectional, quantitative experimental, mixed-methods case study).*
Research sample	*State the research sample (e.g. Harvard university undergraduates, villagers in rural India) and provide relevant demographic information (e.g. age, sex) and indicate whether the sample is representative. Provide a rationale for the study sample chosen. For studies involving existing datasets, please describe the dataset and source.*
Sampling strategy	*Describe the sampling procedure (e.g. random, snowball, stratified, convenience). Describe the statistical methods that were used to predetermine sample size OR if no sample-size calculation was performed, describe how sample sizes were chosen and provide a rationale for why these sample sizes are sufficient. For qualitative data, please indicate whether data saturation was considered, and what criteria were used to decide that no further sampling was needed.*
Data collection	*Provide details about the data collection procedure, including the instruments or devices used to record the data (e.g. pen and paper, computer, eye tracker, video or audio equipment) whether anyone was present besides the participant(s) and the researcher, and whether the researcher was blind to experimental condition and/or the study hypothesis during data collection.*
Timing	*Indicate the start and stop dates of data collection. If there is a gap between collection periods, state the dates for each sample cohort.*
Data exclusions	*If no data were excluded from the analyses, state so OR if data were excluded, provide the exact number of exclusions and the rationale behind them, indicating whether exclusion criteria were pre-established.*
Non-participation	*State how many participants dropped out/declined participation and the reason(s) given OR provide response rate OR state that no participants dropped out/declined participation.*
Randomization	*If participants were not allocated into experimental groups, state so OR describe how participants were allocated to groups, and if allocation was not random, describe how covariates were controlled.*

Ecological, evolutionary & environmental sciences study design

All studies must disclose on these points even when the disclosure is negative.

Study description	*We conducted a whole-ecosystem mercury loading and abatement study (Mercury Experiment To Assess Atmospheric Loading In Canada And the United States; METAALICUS) to directly examine how quickly methylmercury concentrations in fish respond to reductions in inorganic mercury pollution. We added environmentally-relevant amounts of isotope-enriched inorganic mercury to a boreal lake and its catchment for 7 years, then ceased all isotopic inorganic mercury additions to simulate a reduction in mercury*

loading. The lake, upland, and wetland areas of the catchment each received different isotopes, which we quantified (in addition to ambient mercury) in water, sediments and biota of the study lake during the addition phase and for 8 years following cessation of loading. We focus our analyses of fish methylmercury concentrations on three dominant species - yellow perch, northern pike, and lake whitefish - each important in commercial, subsistence and recreational fisheries. Temporal changes in ambient methylmercury concentrations were also monitored for yellow perch and northern pike in a nearby reference lake, consistent with standard whole-ecosystem study designs.

Research sample	Research samples were taken to quantify how different compartments of the lake responded to changes in inorganic mercury loading, and in turn how these compartments influenced fish methylmercury concentrations. Samples were taken during the open-water season and represent annual time-integrated estimates of isotopic and ambient methylmercury concentrations in water, the upper 2 cm of lake sediments, and the dominant invertebrate prey for fish (zooplankton, Chaoborus and chironomids). The dominant fish species in the lake were collected each autumn. We monitored young age classes of small-bodied fishes (yellow perch and blacknose shiner) and older age classes of large-bodied fish species (northern pike and lake whitefish).
Sampling strategy	Sampling in a small lake required careful consideration of potential disturbance to the lake (i.e., benthic and sediment sampling) and fish populations. We conducted large-scale pilot mesocosm studies prior to the whole-ecosystem experiment to inform sampling design and sample size. For small fish species, we collected only the youngest age classes, which are typically the most abundant, to avoid depleting these populations. We developed a non-destructive biopsy technique for large fish so that they could be sampled for methylmercury and returned to the lake. Many of the large fish were biopsied multiple times over the 15 year study which informed our understanding of methylmercury uptake and loss in individual fish. We collected samples of yellow perch and northern pike from a nearby reference lake to monitor annual changes in methylmercury concentrations of an undisturbed system.
Data collection	Data were collected over a period of 15 years (2001-2015) by Principal Investigators, graduate students, biologists/technicians, and research assistants. Data collection methods for each sample type are described in detail in the Methods. Briefly, water samples were collected by pump and filtered through in-line cartridges (tubing and filter apparatus were acid-cleaned Teflon). Sediment cores were collected by hand by divers and through use of a box corer. Zooplankton samples were collected by vertical tows of plankton nets through the water column. Benthic invertebrate samples were collected using an Ekman dredge. Fish samples were collected by trap net, gill net, pole seine net, and angling. All samples were processed using clean techniques for trace metals using stainless steel or Teflon tools. Mercury concentrations were quantified using multi-collector inductively-coupled plasma mass spectrometry (ICP-MS). Methylmercury was analysed in all samples, except for fish muscle tissue, where total mercury was quantified. For a subset of small-bodied fish we determined that most (>90%) of the isotopic and ambient mercury is methylmercury. Samples of certified reference materials were subjected to the same procedures; measured mercury concentrations in the reference materials were not statistically different from certified values.
Timing and spatial scale	Annual sampling frequency varied by compartment (e.g., bi-weekly or monthly for water, zooplankton; annually for fish) over the 15 year period (2001-2015). Sampling for water, sediments, zooplankton and invertebrates occurred during the most productive time of year (open-water season), at a frequency consistent with other whole-ecosystem studies. We consistently collected small- and large-bodied fish species annually from Lake 658 in the autumn months (September-November) as this represents the end of the growing season for north temperate fish species. All samples presented in this study were collected from within the experimental lake (8.4 ha surface area), and from a nearby reference lake.
Data exclusions	A year class failure of yellow perch resulted in a single young-of-year (YOY) collected in 2008 and no age 1+ fish in 2009. Data for the single YOY perch (n=1) captured in 2008 is not presented. A single northern pike biopsy was removed from all analyses due to unreliable mercury data.
Reproducibility	Results of whole ecosystem-scale experiments are rarely exactly reproducible. Results depend on a suite of ecosystem-specific conditions (e.g. for mercury, amount of associated wetland area or hypolimnetic anoxia could influence results), in addition to abiotic factors (e.g. temperature and precipitation) that can vary from year to year. Similar findings from smaller-scale pilot studies at the ELA (in different uplands, wetlands, and lakes) indicate that the findings from the METAALICUS project are generally reproducible.
Randomization	Randomization was not required. Fish samples were collected annually in autumn months from throughout the lake and were grouped in our analyses according to year. Water, sediment, invertebrate, and small-bodied fish (when considered as prey for northern pike) samples collected throughout each year were averaged to represent the mean methylmercury concentration in that compartment each year, as described in the Methods.
Blinding	Blinding was not necessary for this study because fish captures were random as was the selection of a subset of small fish for mercury concentration analyses.

Did the study involve field work? ☒ Yes ☐ No

Field work, collection and transport

Field conditions	Field data collection occurred over a period of 15 years under a variety of field conditions throughout the open water season. The conditions did not influence data or sample collection methods. Annual air temperature from May through October at the ELA averaged 13.9 °C (range: 12.2-15.0 °C) with a range of observed temperatures between -10 °C and 35.5 °C. Cumulative annual precipitation averaged 537 mm (range: 389-734 mm) during the study. The application of enriched stable mercury isotopes to upland and wetland areas was achieved through crop-duster airplane and was undertaken each spring (if possible) just prior to a light rainfall event to simulate wet-deposition of mercury.
Location	Lake 658 (lake and watershed) is located in northwestern Ontario, Canada (49° 43’ 95” N, 93° 44’ 20” W; elevation 371 m). Upland catchment is 41.2 ha, wetland is 1.7 ha, and lake surface is 8.4 ha. Lake 658 is a double-basin lake with a maximum depth of 13 m. The reference lake (Lake 240; 44 ha) is located ~9 km due south of Lake 658.
Access & import/export	All research was conducted at the Experimental Lakes Area (ELA), a remote and pristine region of northwestern Ontario, Canada, where 58 lakes and their watersheds have been set aside for research on anthropogenic impacts to freshwater ecosystems. Lake 658

and the reference lake (Lake 240) are designated ELA research lakes. From 1968 to 2014, ELA was a Federal Government facility. In 2014, ownership of ELA was transferred to the International Institute for Sustainable Development (now IISD-ELA). All research presented here was approved by provincial and federal authorities (see below). An annual License to Collect Fish for Scientific Purposes was awarded each year as required by the provincial government.

Disturbance

Whole-ecosystem research examining the effects of various human impacts on the environment has been ongoing at ELA since its inception in 1968. The METAALICUS project, as with all others conducted at ELA, was reviewed and approved by the ELA Research Advisory Board (Fisheries and Oceans Canada), Ontario Ministry of Natural Resources and Forestry, Ontario Ministry of the Environment and Climate Change, and Ontario Parks. The many layers of review and approval are in place to ensure that only important, legitimate, high quality science projects are undertaken at ELA. These agreements also ensure that lakes are returned to their natural state following manipulation.

In this project, the small amount of mercury added to the lake and watershed over 7 years (~1 teaspoon) did not pose a human health threat. Lake 658 itself is closed to recreational fishing and bait harvest, such that none of the fish from the lake were handled or removed by the public. A fence was installed at the outflow to prevent the the movement of fish between the downstream lake and Lake 658 for the duration of the study (as required by OMNRF). In accordance with monitoring requirements, a small number of fish in the downstream lake were collected annually.

Reporting for specific materials, systems and methods

We require information from authors about some types of materials, experimental systems and methods used in many studies. Here, indicate whether each material, system or method listed is relevant to your study. If you are not sure if a list item applies to your research, read the appropriate section before selecting a response.

Materials & experimental systems

n/a	Involved in the study	
☒	☐	Antibodies
☒	☐	Eukaryotic cell lines
☒	☐	Palaeontology and archaeology
☐	☒	Animals and other organisms
☒	☐	Human research participants
☒	☐	Clinical data
☒	☐	Dual use research of concern

Methods

n/a	Involved in the study	
☒	☐	ChIP-seq
☒	☐	Flow cytometry
☒	☐	MRI-based neuroimaging

Antibodies

Antibodies used

Describe all antibodies used in the study; as applicable, provide supplier name, catalog number, clone name, and lot number.

Validation

Describe the validation of each primary antibody for the species and application, noting any validation statements on the manufacturer's website, relevant citations, antibody profiles in online databases, or data provided in the manuscript.

Eukaryotic cell lines

Policy information about cell lines

Cell line source(s)

State the source of each cell line used.

Authentication

Describe the authentication procedures for each cell line used OR declare that none of the cell lines used were authenticated.

Mycoplasma contamination

Confirm that all cell lines tested negative for mycoplasma contamination OR describe the results of the testing for mycoplasma contamination OR declare that the cell lines were not tested for mycoplasma contamination.

Commonly misidentified lines
(See ICLAC register)

Name any commonly misidentified cell lines used in the study and provide a rationale for their use.

Palaeontology and Archaeology

Specimen provenance

Provide provenance information for specimens and describe permits that were obtained for the work (including the name of the issuing authority, the date of issue, and any identifying information). Permits should encompass collection and, where applicable, export.

Specimen deposition

Indicate where the specimens have been deposited to permit free access by other researchers.

Dating methods

If new dates are provided, describe how they were obtained (e.g. collection, storage, sample pretreatment and measurement), where they were obtained (i.e. lab name), the calibration program and the protocol for quality assurance OR state that no new dates are provided.

☐ Tick this box to confirm that the raw and calibrated dates are available in the paper or in Supplementary Information.

Ethics oversight	*Identify the organization(s) that approved or provided guidance on the study protocol, OR state that no ethical approval or guidance was required and explain why not.*

Note that full information on the approval of the study protocol must also be provided in the manuscript.

Animals and other organisms

Policy information about studies involving animals; ARRIVE guidelines recommended for reporting animal research

Laboratory animals	*The study did not involve laboratory animals.*
Wild animals	*All fish were wild strain. In total, 285 yellow perch (Perca flavescens, 145 young-of-year and 140 age-1) and 138 blacknose shiner (Notropis heterolepis, age-1) were captured by pole seine net, small mesh gill net (20 minute set), and hoop net. These fish were euthanized in an overdose bath of tricaine methanesulfonate (0.25 g/L). Most small fish had not reached sexual maturity.*<br><br>*Northern pike (Esox lucius, aged at 2-12 y), lake whitefish (Coregonus clupeaformis, aged at 3-38 y), and white sucker (Catostomus commersonii, not aged) were captured by angling or multi-mesh gill nets (20-30 min set). Fish were anesthetized with tricaine methanesulfonate (0.06 g/L), basic biological information was collected, fish were tagged with a Passive Integrated Transponder (PIT) tag, and a small biopsy of dorsal muscle was collected using a dermal punch which was then sealed with veterinary tissue adhesive (VetBond). Fish were allowed to recover in a tub of fresh lake water (for ~15 min) before being released back into the lake. In total, 690 biopsy muscle samples were collected from 390 fish (238 northern pike, 114 lake whitefish, 38 white sucker) from 2001-2015 in Lake 658; 149 fish (90 northern pike, 38 lake whitefish, 21 white sucker) were biopsied more than once during the 15 year study (2 to 6 biopsies per individual). Sex data were not available for most fish as they were mostly captured outside of their spawning season.*<br><br>*Collection of zooplankton and benthic macroinvertebrates is not regulated or licenced in Ontario. Zooplankton samples were collected by vertical tows of plankton nets through the water column. Benthic invertebrate samples were collected using an Ekman dredge.*
Field-collected samples	*The study did not house samples collected from the field in the lab.*
Ethics oversight	*All work with vertebrate animals was approved by Animal Care Committees (ACC) through the Canadian Council on Animal Care (Freshwater Institute ACC for Fisheries & Oceans Canada, 2001-2013; University of Manitoba ACC for IISD-ELA, 2014-2015). Licenses to Collect Fish for Scientific Purposes were granted annually by the Ontario Ministry of Natural Resources and Forestry.*

Note that full information on the approval of the study protocol must also be provided in the manuscript.

Human research participants

Policy information about studies involving human research participants

Population characteristics	*Describe the covariate-relevant population characteristics of the human research participants (e.g. age, gender, genotypic information, past and current diagnosis and treatment categories). If you filled out the behavioural & social sciences study design questions and have nothing to add here, write "See above."*
Recruitment	*Describe how participants were recruited. Outline any potential self-selection bias or other biases that may be present and how these are likely to impact results.*
Ethics oversight	*Identify the organization(s) that approved the study protocol.*

Note that full information on the approval of the study protocol must also be provided in the manuscript.

Clinical data

Policy information about clinical studies
All manuscripts should comply with the ICMJE guidelines for publication of clinical research and a completed CONSORT checklist must be included with all submissions.

Clinical trial registration	*Provide the trial registration number from ClinicalTrials.gov or an equivalent agency.*
Study protocol	*Note where the full trial protocol can be accessed OR if not available, explain why.*
Data collection	*Describe the settings and locales of data collection, noting the time periods of recruitment and data collection.*
Outcomes	*Describe how you pre-defined primary and secondary outcome measures and how you assessed these measures.*

Dual use research of concern

Policy information about dual use research of concern

Hazards

Could the accidental, deliberate or reckless misuse of agents or technologies generated in the work, or the application of information presented in the manuscript, pose a threat to:

No	Yes	
☐	☐	Public health
☐	☐	National security
☐	☐	Crops and/or livestock
☐	☐	Ecosystems
☐	☐	Any other significant area

Experiments of concern

Does the work involve any of these experiments of concern:

No	Yes	
☐	☐	Demonstrate how to render a vaccine ineffective
☐	☐	Confer resistance to therapeutically useful antibiotics or antiviral agents
☐	☐	Enhance the virulence of a pathogen or render a nonpathogen virulent
☐	☐	Increase transmissibility of a pathogen
☐	☐	Alter the host range of a pathogen
☐	☐	Enable evasion of diagnostic/detection modalities
☐	☐	Enable the weaponization of a biological agent or toxin
☐	☐	Any other potentially harmful combination of experiments and agents

ChIP-seq

Data deposition

☐ Confirm that both raw and final processed data have been deposited in a public database such as GEO.

☐ Confirm that you have deposited or provided access to graph files (e.g. BED files) for the called peaks.

Data access links<br>*May remain private before publication.*	*For "Initial submission" or "Revised version" documents, provide reviewer access links. For your "Final submission" document, provide a link to the deposited data.*
Files in database submission	*Provide a list of all files available in the database submission.*
Genome browser session<br>(e.g. UCSC)	*Provide a link to an anonymized genome browser session for "Initial submission" and "Revised version" documents only, to enable peer review. Write "no longer applicable" for "Final submission" documents.*

Methodology

Replicates	*Describe the experimental replicates, specifying number, type and replicate agreement.*
Sequencing depth	*Describe the sequencing depth for each experiment, providing the total number of reads, uniquely mapped reads, length of reads and whether they were paired- or single-end.*
Antibodies	*Describe the antibodies used for the ChIP-seq experiments; as applicable, provide supplier name, catalog number, clone name, and lot number.*
Peak calling parameters	*Specify the command line program and parameters used for read mapping and peak calling, including the ChIP, control and index files used.*
Data quality	*Describe the methods used to ensure data quality in full detail, including how many peaks are at FDR 5% and above 5-fold enrichment.*
Software	*Describe the software used to collect and analyze the ChIP-seq data. For custom code that has been deposited into a community repository, provide accession details.*

Flow Cytometry

Plots

Confirm that:

- [ ] The axis labels state the marker and fluorochrome used (e.g. CD4-FITC).
- [ ] The axis scales are clearly visible. Include numbers along axes only for bottom left plot of group (a 'group' is an analysis of identical markers).
- [ ] All plots are contour plots with outliers or pseudocolor plots.
- [ ] A numerical value for number of cells or percentage (with statistics) is provided.

Methodology

Sample preparation	*Describe the sample preparation, detailing the biological source of the cells and any tissue processing steps used.*
Instrument	*Identify the instrument used for data collection, specifying make and model number.*
Software	*Describe the software used to collect and analyze the flow cytometry data. For custom code that has been deposited into a community repository, provide accession details.*
Cell population abundance	*Describe the abundance of the relevant cell populations within post-sort fractions, providing details on the purity of the samples and how it was determined.*
Gating strategy	*Describe the gating strategy used for all relevant experiments, specifying the preliminary FSC/SSC gates of the starting cell population, indicating where boundaries between "positive" and "negative" staining cell populations are defined.*

- [ ] Tick this box to confirm that a figure exemplifying the gating strategy is provided in the Supplementary Information.

Magnetic resonance imaging

Experimental design

Design type	*Indicate task or resting state; event-related or block design.*
Design specifications	*Specify the number of blocks, trials or experimental units per session and/or subject, and specify the length of each trial or block (if trials are blocked) and interval between trials.*
Behavioral performance measures	*State number and/or type of variables recorded (e.g. correct button press, response time) and what statistics were used to establish that the subjects were performing the task as expected (e.g. mean, range, and/or standard deviation across subjects).*

Acquisition

Imaging type(s)	*Specify: functional, structural, diffusion, perfusion.*
Field strength	*Specify in Tesla*
Sequence & imaging parameters	*Specify the pulse sequence type (gradient echo, spin echo, etc.), imaging type (EPI, spiral, etc.), field of view, matrix size, slice thickness, orientation and TE/TR/flip angle.*
Area of acquisition	*State whether a whole brain scan was used OR define the area of acquisition, describing how the region was determined.*

Diffusion MRI ☐ Used ☐ Not used

Preprocessing

Preprocessing software	*Provide detail on software version and revision number and on specific parameters (model/functions, brain extraction, segmentation, smoothing kernel size, etc.).*
Normalization	*If data were normalized/standardized, describe the approach(es): specify linear or non-linear and define image types used for transformation OR indicate that data were not normalized and explain rationale for lack of normalization.*
Normalization template	*Describe the template used for normalization/transformation, specifying subject space or group standardized space (e.g. original Talairach, MNI305, ICBM152) OR indicate that the data were not normalized.*
Noise and artifact removal	*Describe your procedure(s) for artifact and structured noise removal, specifying motion parameters, tissue signals and physiological signals (heart rate, respiration).*

Volume censoring	*Define your software and/or method and criteria for volume censoring, and state the extent of such censoring.*

Statistical modeling & inference

Model type and settings	*Specify type (mass univariate, multivariate, RSA, predictive, etc.) and describe essential details of the model at the first and second levels (e.g. fixed, random or mixed effects; drift or auto-correlation).*
Effect(s) tested	*Define precise effect in terms of the task or stimulus conditions instead of psychological concepts and indicate whether ANOVA or factorial designs were used.*

Specify type of analysis: ☐ Whole brain ☐ ROI-based ☐ Both

Statistic type for inference<br>(See Eklund et al. 2016)	*Specify voxel-wise or cluster-wise and report all relevant parameters for cluster-wise methods.*
Correction	*Describe the type of correction and how it is obtained for multiple comparisons (e.g. FWE, FDR, permutation or Monte Carlo).*

Models & analysis

n/a	Involved in the study
☐	☐ Functional and/or effective connectivity
☐	☐ Graph analysis
☐	☐ Multivariate modeling or predictive analysis

Functional and/or effective connectivity	*Report the measures of dependence used and the model details (e.g. Pearson correlation, partial correlation, mutual information).*
Graph analysis	*Report the dependent variable and connectivity measure, specifying weighted graph or binarized graph, subject- or group-level, and the global and/or node summaries used (e.g. clustering coefficient, efficiency, etc.).*
Multivariate modeling and predictive analysis	*Specify independent variables, features extraction and dimension reduction, model, training and evaluation metrics.*
